# Identification of apple leaf disease via novel attention mechanism based convolutional neural network

**DOI:** 10.3389/fpls.2023.1274231

**Published:** 2023-10-18

**Authors:** Hebin Cheng, Heming Li

**Affiliations:** School of Intelligence Engineering, Shandong Management University, Jinan, China

**Keywords:** apple leaf disease, classification, deep learning, attention mechanism, multi-scale feature extraction

## Abstract

**Introduction:**

The identification of apple leaf diseases is crucial for apple production.

**Methods:**

To assist farmers in promptly recognizing leaf diseases in apple trees, we propose a novel attention mechanism. Building upon this mechanism and MobileNet v3, we introduce a new deep learning network.

**Results and discussion:**

Applying this network to our carefully curated dataset, we achieved an impressive accuracy of 98.7% in identifying apple leaf diseases, surpassing similar models such as EfficientNet-B0, ResNet-34, and DenseNet-121. Furthermore, the precision, recall, and f1-score of our model also outperform these models, while maintaining the advantages of fewer parameters and less computational consumption of the MobileNet network. Therefore, our model has the potential in other similar application scenarios and has broad prospects.

## 1 Introduction

Apple is one of the most popular and widely grown fruits worldwide and has been cultivated by humans for over 2000 years. Apple fruit is rich in vitamins and minerals, with high nutritional value, and is an indispensable part of a healthy diet. However, the production of apples is also hindered by various diseases, which can seriously affect the yield and quality of apples. Traditional plant disease identification, management, and prevention rely on the experience of farmers and local agricultural technicians. When these measures are insufficient, it is impossible to accurately identify the diseases and timely intervene, causing great losses to apple production. In the past decade, with the continuous development and progress of machine learning (ML), especially the advancement of deep learning (DL) technology, the accuracy of identifying leaf diseases has been continuously improved, paving the way for more efficient and real-time disease detection. Kamilaris and Prenafeta-Boldú (2018); Pardede et al. (2018).

The disease recognition of plant leaves is essentially an image classification problem that requires accurate capture of disease features, comparison with other types of diseases, and classification. Traditional ML methods typically use image processing and classifier for plant disease recognition. The image processing methods include extracting the color and texture of disease spots through grayscale values or performing pixel-level segmentation of disease spots. Deng et al. (2019) Support vector machine (SVM), Mokhtar et al. (2015) k-means clustering, Naive Bayes, etc. Ma et al. (2018) are most widely used classifier. Tradition ML has good recognition accuracy for diseases with certain characteristics. Singh et al. (2016) However, the generalization of these methods is poor, limited by the inability to recognition of nonlinear data and the difficulty of feature extraction. Once the processing object changes, the model cannot perform reasonable classification.

Convolutional neural network (CNN) automatically extracts features directly from the original image, greatly improving the efficiency of image classification. Therefore, with the emergence of CNN, especially the success of AlexNET in the competition of ImageNet LSVRC-2010, Krizhevsky et al. (2017); Shin et al. (2021) a series of DL models have been proposed, such as GoogleNet, Inception, VGG, ResNet, DenseNet, etc. Not surprisingly, these DL networks have also been used by researchers in plant disease detection. For example, Fuentes et al. present a deep-learning-based model to detect diseases and pests in tomato plants. They proposed a two-stage model which combines the meta-architecture (faster R-CNN) with feature extractors such as VGG and ResNet. Their system can effectively recognize nine different types of diseases and pests in complex surroundings. Fuentes et al. (2017) Khan, et al. utilized a hybrid method -a segmentation method which followed pre-trained deep models to achieve the classification accuracy of 98.60% on public datasets. Khan et al. (2018) Ferentinos compared some DL networks such as AlexNet, GoogLeNet, and VGG et al. and reported a 99.53% accuracy with VGG16 on the extended PlantVillage dataset. Ferentinos (2018) Arsenovic et al. proposed a novel two-stage architecture of a neural network which focused on a real environment plant disease classification. Their model achieved an accuracy of 93.67%. Arsenovic et al. (2019) Too, et al. compared many DL architecture and evaluated the best performance of DenseNet-121 in the experiment. Too et al. (2019). Shoaib et al. utilized the Inception Net model in the research work. For the detection and segmentation of disease-affected regions, two state-of the-art semantic segmentation models, i.e., U-Net and Modified U-Net, are utilized in their work too. Shoaib et al. (2022) At the same time, in the segmented field of apple leaf disease detection, a number of research achievements have also emerged. Hasan et al. (2022) For example Jiang et al. proposed an INAR-SSD (incorporating Inception module and Rainbow concatenation) model that achieves a detection accuracy of 78.80% mean Average Precision (mAP) on the apple leaf disease dataset, while maintaining a rapid detection speed of 23.13 frames per second (FPS) Jiang et al. (2019). Sun et al, proposed a novel MEAN-SSD (Mobile End AppleNet based SSD algorithm) model, which can achieve the detection performance of 83.12% mAP and a speed of 12.53 FPS. Sun et al. (2021).

MobileNet is a lightweight network proposed by Google and is widely used by researchers. Howard et al. (2017); Wang et al. (2021); Xiong et al. (2020) In MobileNet v1, depthwise separable convolution was first proposed, which combines depthwise convolution and pointwise convolution in the module. The computational complexity was successfully reduced to 1/9 of that of ordinary convolution. Therefore it greatly reduces computational parameters and improves the speed of model computation. Sandler et al. (2018) In MobileNet v2, the interest manifold is captured by inserting a linear bottleneck in the convolution module instead of the original nonlinear activation function. Kavyashree and El-Sharkawy (2021) The researchers also proposed the inverted residual structure, which expands dimensions through an expansion layer. The depthwise separable convolutions are used to extract features, and projection layers are used to compress data, making the network smaller again. Through this structure, the dimensionality and computational speed of convolutions are balanced, enhancing the performance of the network. In MobileNet v3, the Squeeze-and-Excitation (SE) attention mechanism is further introduced. The SE module is added to the inverted residual structure, and the activation function is updated. Howard et al. (2019) Compared to MobileNet v2, the computational speed and accuracy of MobileNet v3 have been further improved.

In recent years, more Transfer learning (TL) strategies are used in DL. Chen et al. (2020); Coulibaly et al. (2019) These DL models require a large amount of labeled data to achieve good performance. However, in many real-world scenarios, obtaining such a large amount of labeled data may be expensive, time-consuming, or impractical. TL enables the utilization of pre-existing large-scale datasets, such as ImageNet or COCO data sets, and transfers the knowledge obtained from them to the target tasks. On the other hand, DL models consist of multiple layers that learn the hierarchical representation of data. Early layers capture general low-level features (such as edges and textures), while later layers capture high-level semantic features. By using TL, we can reuse low-level and intermediate features learned from pre-trained models as feature extractors. This reduces the need to train these layers from scratch and allows us to focus on training only the top layers specific to our tasks. In the training process of our model, we also adopted the method of TL and achieved very good results.

In this article, we propose a deep learning model named mobileNet-MFS, where MFS is the abbreviation for multi-fused spatial. The main contributions of our work include:

1. A novel fused spatial channel attention (FSCA) mechanism is proposed, which considers both channel and spatial connections of the input layer. We use it to replace the Squeeze-and-Excitation(SE) attention mechanism in the MobileNet v3 architecture and significantly improve the performance of the model.
2. In order to include multi-dimensional information in neural networks, a multi-scale feature extraction module was applied in our network, which fused image features through convolutions of different dimensions. Research has shown that this module has successfully improved the model’s accuracy.
3. Our proposed MobileNet-MFS model has better performance than the original version of MobileNet v3, demonstrating advantages in accuracy, computational speed, parameter size, and other aspects compared to MobileNet VIT, EffientNet, ShuffleNet, DenseNet in diagnosing apple leaf diseases.

## 2 Methodology

### 2.1 Network architechture

**Figure 1:**
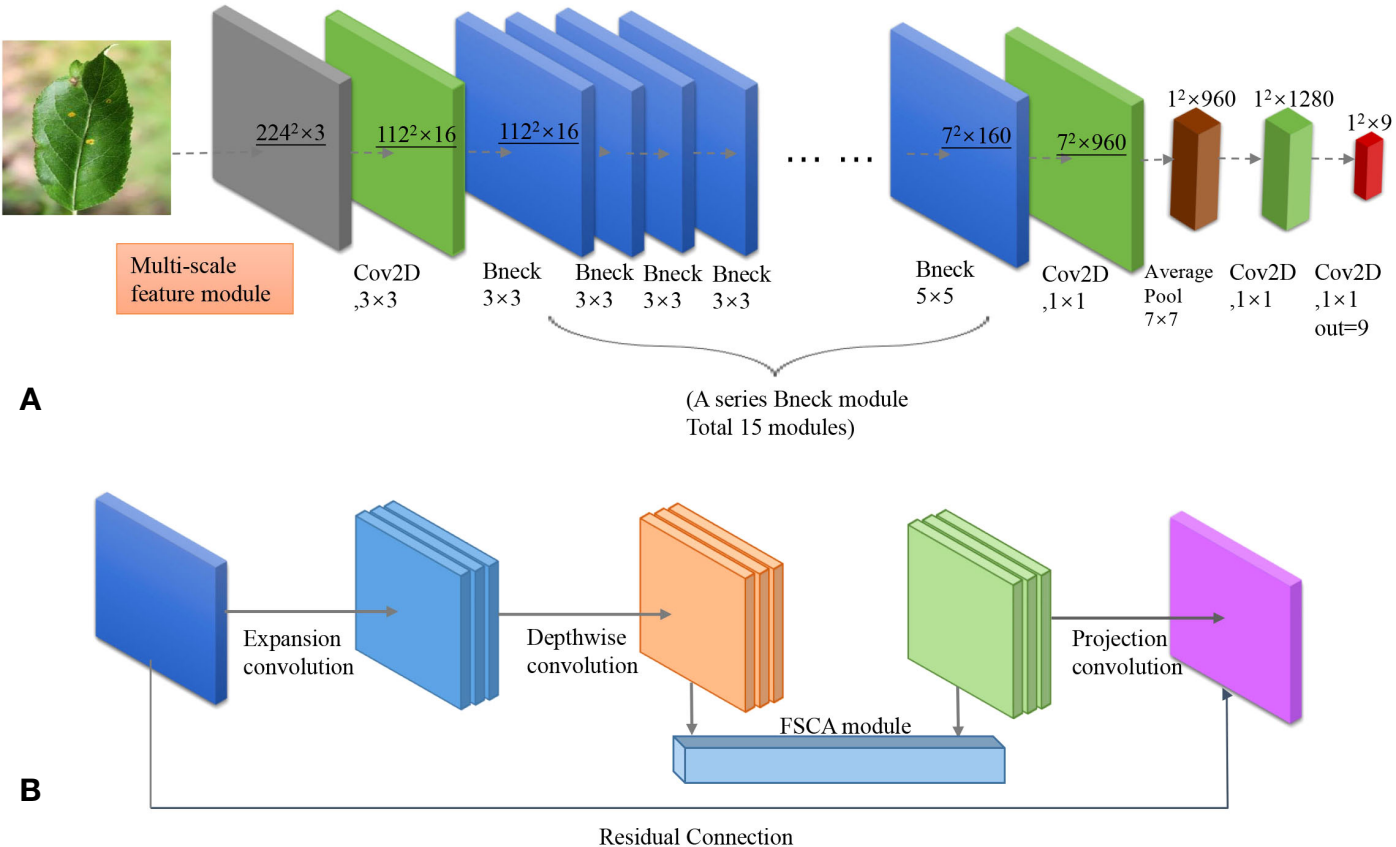
**(A)** Network structure of MobileNet-MFS. **(B)** Detailed composition of a single bottleneck module.

The network architecture of our model(MobileNet-MFS) is shown in **Figure 1A**. The design of the model inherits the main modules of MobileNet v3, but in order to obtain better diagnostic efficacy, many modifications were also made to the model. The main body of the model is consistent with MobileNet v3, which consists of a two-dimensional convolutional layer, a series of bottleneck layers with different dimensions, a two-dimensional convolutional layer, a pooling layer, and a one-dimensional convolutional layer in sequence. Through this series of modules, feature information on plant disease-affected areas is extracted, and diseases are classified into 9 types through 1×1 convolution. However, at the front end of the model, in order to further explore the feature information that cannot be captured in the original MobileNet v3, we introduced a multi-scale feature extraction module. The most important change is that we have proposed a new FSCA attention mechanism to replace the SE attention mechanism module used in MobileNeT v3. The FSCA mechanism will be explained in detail in the following chapters.

As shown in **Figure 1B**, in MobileNet-MFS, the most basic module is the bottleneck layer, which is composed of an inverted residual network containing depthwise separated convolutions. It replaces the standard convolution operation with a depthwise convolution followed by a pointwise convolution. This reduces computational complexity and model size while maintaining accuracy. In addition to depthwise separated convolution, the bottleneck layer also includes expansion convolution, which mainly serves to increase the number of channels in the input feature map using a 1x1-sized convolutional kernel. Projection convolution is a 1x1 convolutional kernel with a significantly smaller number of output channels than the input channels, thus achieving the goal of limiting the size of the model. When the input and output channels are the same, a residual network can be used. The bottleneck layer of the inverted residual structure formed by the above convolution operations is finally activated using ReLU or h-swish function.

### 2.2 Attention module

Although CNN is very powerful in image expression, they are deficient in expressing spatial information. Therefore, the attention mechanism has been introduced into MobileNet v3, which can improve the learning ability of the model by assigning weights to images. In the original version of MobileNet v3, the SE attention module is placed in the middle of the bottleneck layer, Hu et al. (2020) giving an updated set of weight values through two fully connection layers and the activation function. However, the SE attention module only cares about the dependencies between channels and ignores location information, which is crucial for generating spatially selective attention maps. Therefore, we propose our FSCA attention mechanism to replace the SE module.

**Figure 2:**
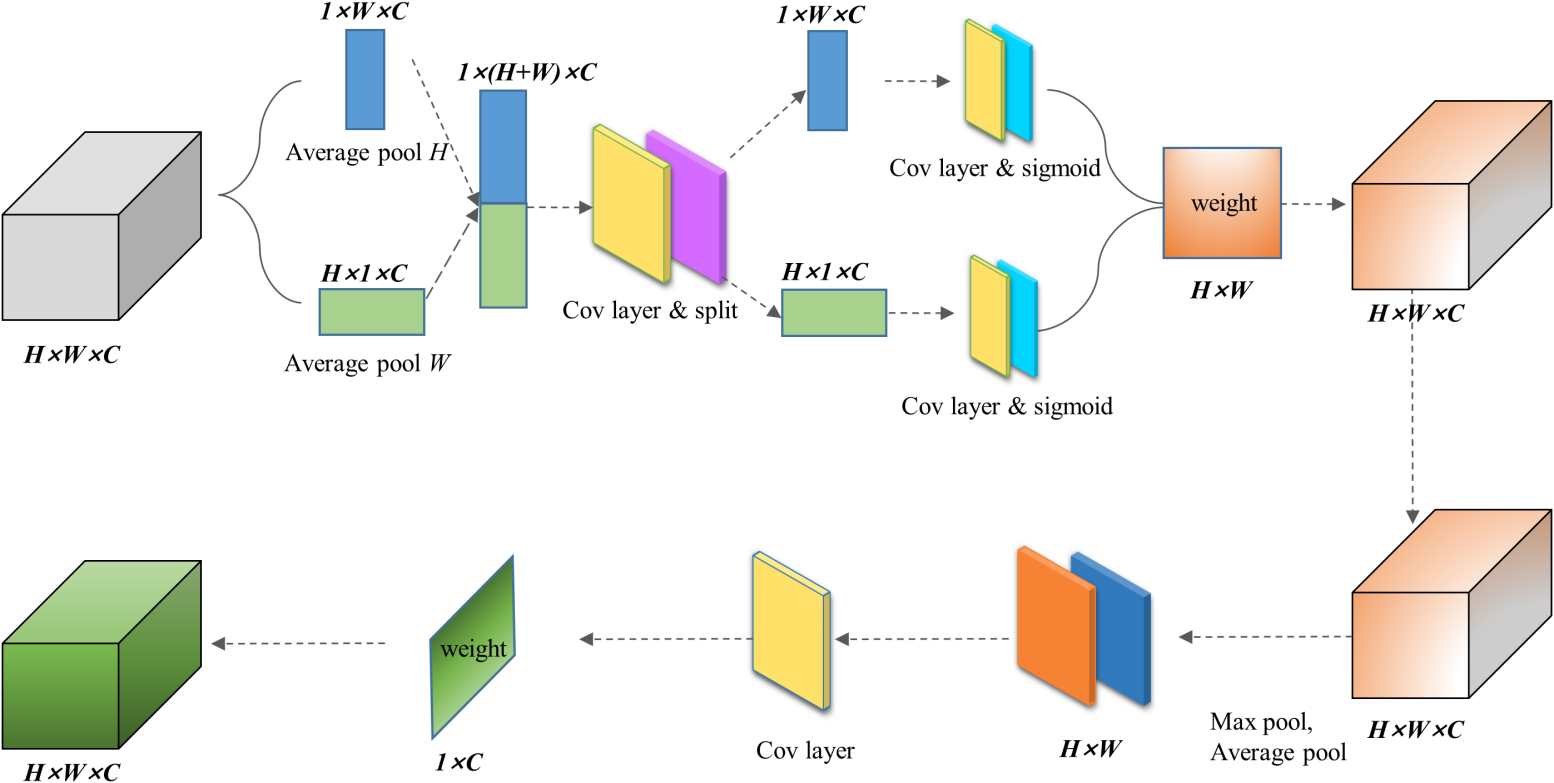
Network architecture of FSCA attention mechanism module.

The FSCA attention mechanism considers both spatial and channel information of the input layer, thus more effectively guiding the model to focus on effective positions in the image. As shown in **Figure 2**, the FSCA attention mechanism consists of two concatenated modules. The first module mainly focuses on aggregating features in the spatial directions of X and Y. By averaging pooling in the X and Y directions and performing concat operation, a 1 × (H+W) × C dimensional array is obtained. Furthermore, we normalized the array through convolution, separated it, and activated it with a sigmoid function to obtain a set of weights containing information in the X and Y directions. Afterward, the weights are multiplied with the original data to obtain a set of directional perception feature layers. These transformations allow the attention module to capture long-term dependencies along one spatial direction and preserve precise positional information along another spatial direction, which helps the network locate interested targets more accurately.

The second module focuses on channel attention. In this module, we will take the maximum and average values of the input feature layers on the channels of each feature point. Afterward, we stack these two values and adjust the number of channels using a convolution with a channel count of 1. Then, we take a sigmoid function and obtain the weights of each feature point in the input feature layer (between 0 and 1). After obtaining this weight, we multiply it by the original input feature layer.

By concatenating and multiplying the two steps, we obtain our FSCA attention mechanism, which focuses on both the X and Y dimensions of input and the fusion of information of channels. Therefore, the obtained results are more comprehensive. Since our attention mechanism fused both spatial and channel information, we named it FSCA attention mechanism, which references CBAM Woo et al. (2018) and CA Hou et al. (2021) attention mechanism. In the following experiments, it was demonstrated that the FSCA mechanism helped our model better identify the characteristics of apple leaf diseases.

### 2.3 Multi-scale feature extraction

For apple leaf diseases, there are two main characteristics that are not easily extracted by machines. One is that there is a significant difference in the size of the disease on the leaves, such as Powdery Mill Draw and Grey spot lesions. Another type of disease is that its color or other details may vary depending on the scope of the disease, such as Grey spot and Rust lesions.

The above features involve dimensions of different sizes and are not easily captured by MobileNet V3, which mainly uses 3 × 3 and 5 × 5 convolution operations. In order to enable the machine to capture more features from different dimensions, Li et al. (2020) we have added a multi-scale feature extraction module to the front end of the input layer.

**Figure 3:**
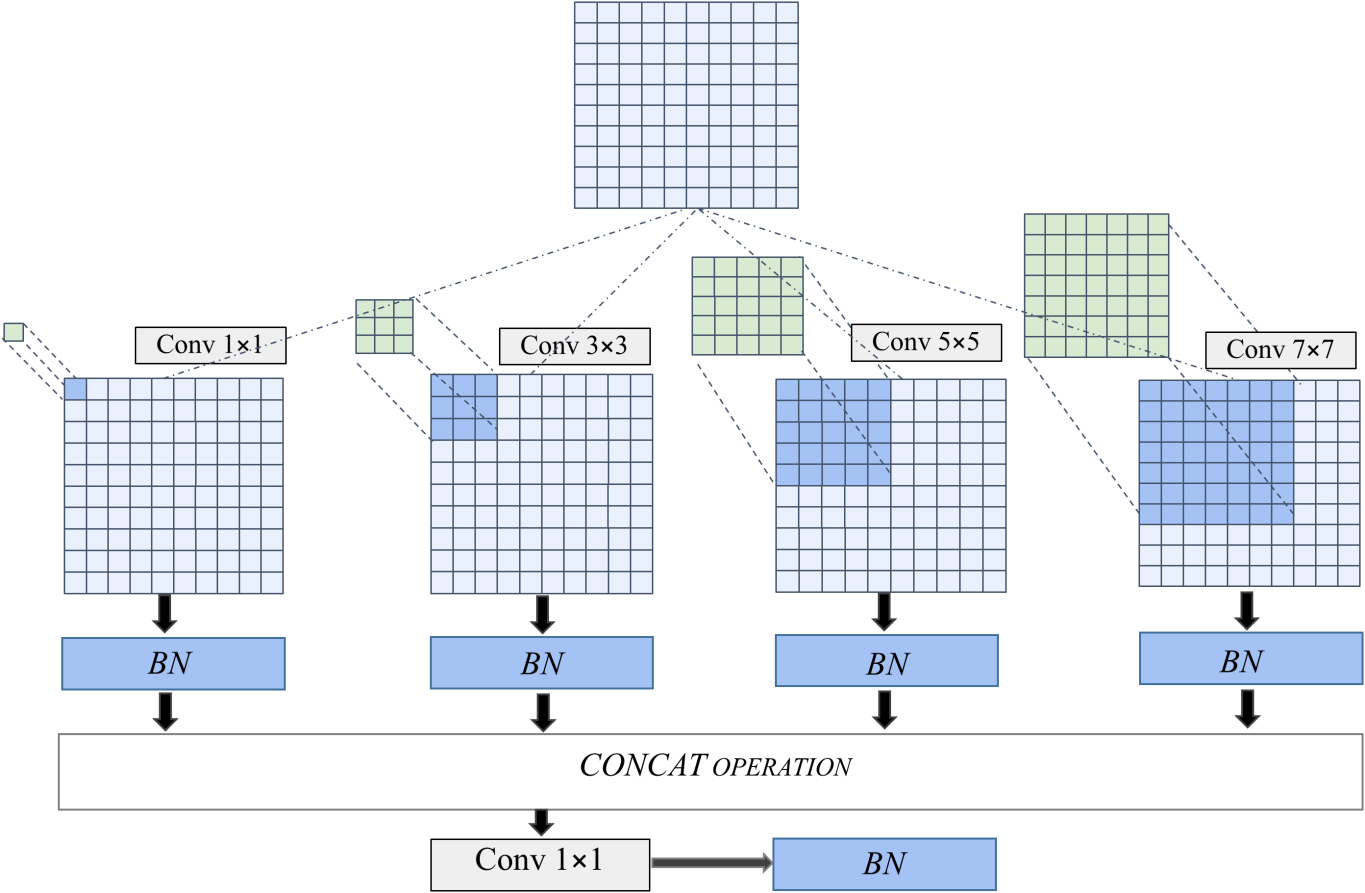
Compositions of multi-feature extraction module.

The structure of this module is shown in **Figure 3**. Four dimensions of convolution: 1 × 1, 3 × 3, 5 × 5, and 7 × 7 were applied in the module. After the image is convoluted, it is merged into a new feature map and then placed ahead of the network. Through such feature extraction, the accuracy of disease classification was improved.

## 3 Experimental results

### 3.1 Dataset

The images of apple leaves were collected from both laboratory and outdoor environments, with a total of eight diseases. These leaves were divided into nine categories, and each photo was labeled with the disease type. Our data mainly comes from PlantVillage, PPCD2020, PPCD2021, and ATLDSD datasets. PlantVillage is mainly from laboratory environments, while images from the PPCD2020 and PPCD2021 are collected in natural environments. The total number of samples is 15250, including 12204 for the training set and 3046 for the testing set. The sample ratio for the training and testing sets is 4:1.

**Figure 4:**
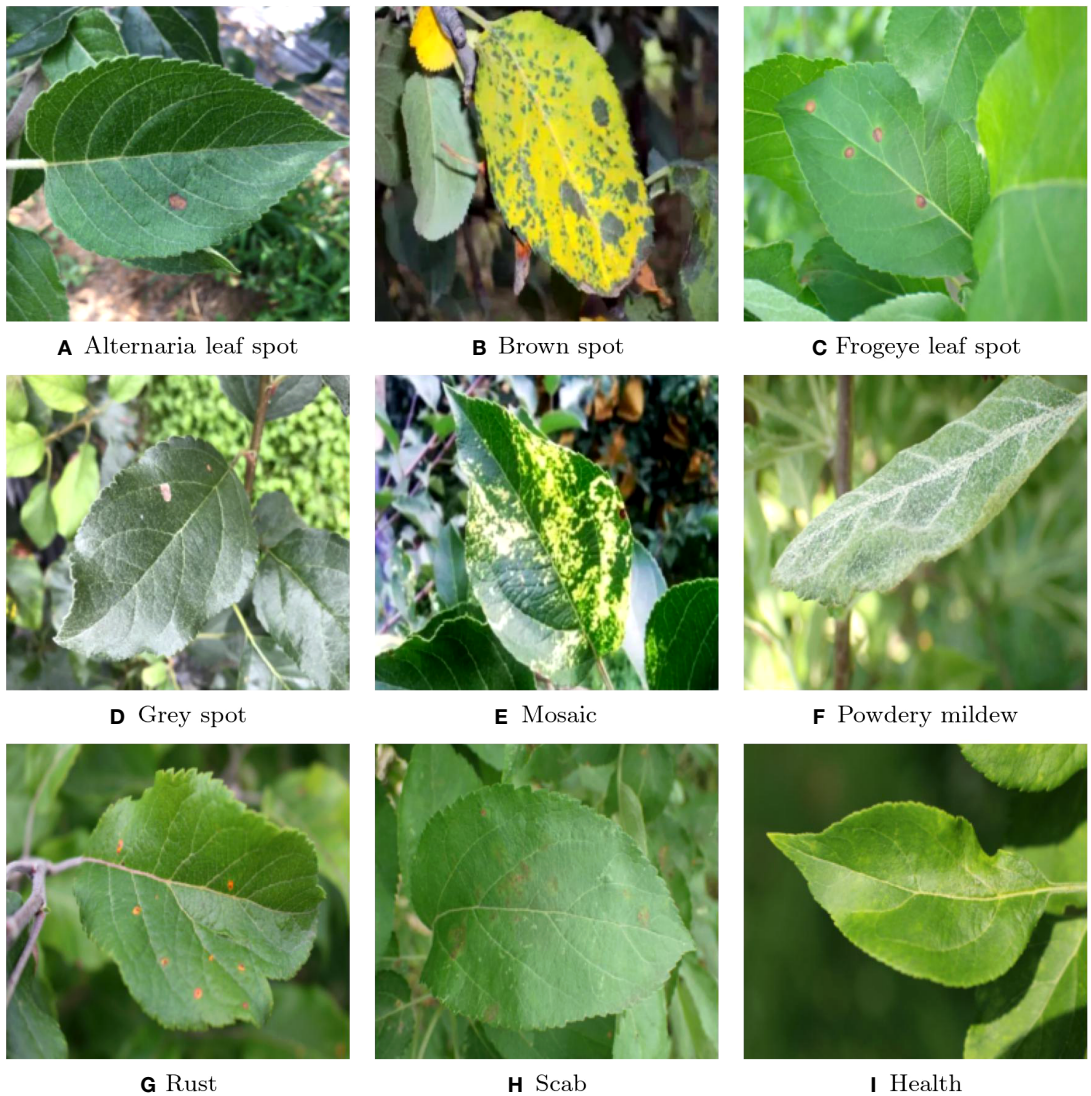
Classification of the samples: **(A)** Alternaria leaf spot; **(B)** Brown spot; **(C)** Frogeye leaf spot; **(D)** Grey spot; **(E)** Mosaic; **(F)** Powdery mildew; **(G)** Rust; **(H)** Scab; **(I)** Health.

As shown in **Figure 4**, there are a total of eight apple diseases in our sample, namely Alternaria leaf spot, Brown spot, Frogeye leaf spot, Grey spot, Mosaic, Powdery Mildew, Rust and Scab. The number of samples was collected in **Table 1**. Both Brown spot and Mosaic form large spots on the leaves, but the former will first cause the diseased parts of the leaves to turn yellow in a large area. Powdery Mildew can turn the veins of the leaves white and stain the leaves with white spots. Many other plants also suffer from similar diseases, such as strawberries. Other diseases can cause various types of spots on the leaves, such as Rust causing red spots on the leaves, while Gray spots causing gray spots, and Frogeyes causing yellow-brown spots on the center, similar to those on the outer ring of a frog’s eye. In order to distinguish these different types of spots, neural networks need to first be able to capture these spots and further distinguish the different features of color and shape in the spots.

**Table 1:** Number of samples from different diseases.

Types	Training Sample	Test Sample	Total Sample	Total (data augumation)
Alternaria leaf spot	526	131	657	1578
Brown spot	354	88	442	1062
Frogeye leaf spot	2544	635	3179	7632
Grey spot	285	71	356	855
Health	704	175	879	2112
Mosaic	316	79	395	948
Powdery mildew	947	236	1183	2841
Rust	2202	550	2752	6606
Scab	4326	1081	5407	12978
Total Number	12204	3046	15250	36612

### 3.2 Evaluation metric

Accuracy is the most commonly used indicator, which represents the proportion of the true value of a model in the overall population. However, measuring the quality of a model cannot be solely based on accuracy. Some other indicators also reflect the quality of the model. For example, precision focuses on the model’s ability to avoid false positives, while recall focuses on the model’s ability to identify all positive instances. At the same time, when the dataset of the model is imbalanced, the f1-score balances the results of recall and precision, which better reflects the advantages and disadvantages of the model. The area under curve (AUC) shows the trade-off between the true positive rate and the false positive rate. Higher AUC values indicate better discriminability of the model. Therefore, accuracy is used with other performance metrics like precision, recall, f1-Score, and AUC. The definition of accuracy is:


(1) $$Accuracy=\frac{TP+FN}{TP+FP+TN+FN}$$

where TN = true negative, FN = false negative, TP = true positive, and FP = false positive.

The expression of precision, recall, and f1-score are equations (2–4), respectively.


(2) $$Precision=\frac{TP}{TP+FP}$$

(3) $$Recall=\frac{TP}{TP+FN}$$

(4) $$F1score=\frac{2\times Precision\times Recall}{Precision+Recall}$$

## 4 Result

### 4.1 Accuracy and Loss

**Figure 5:**
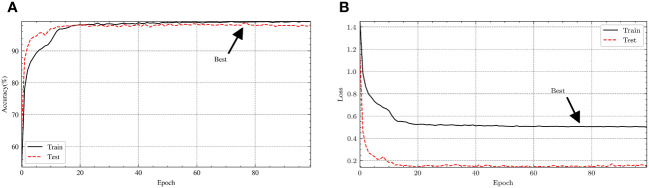
The **(A)** accuracy and **(B)** loss curve of the experiment.

The accuracy and loss values of the model are shown in **Figure 5**. By analyzing the images, we can conclude that the accuracy of training and testing has improved to over 97% after 20 epochs. For the training data, the loss is around 0.5, while for the test data, the loss stabilizes below 0.1 after 20 epochs. When epochs approach 80, the model achieved a maximum accuracy of 98.7%.

### 4.2 Confusion matrix

**Figure 6:**
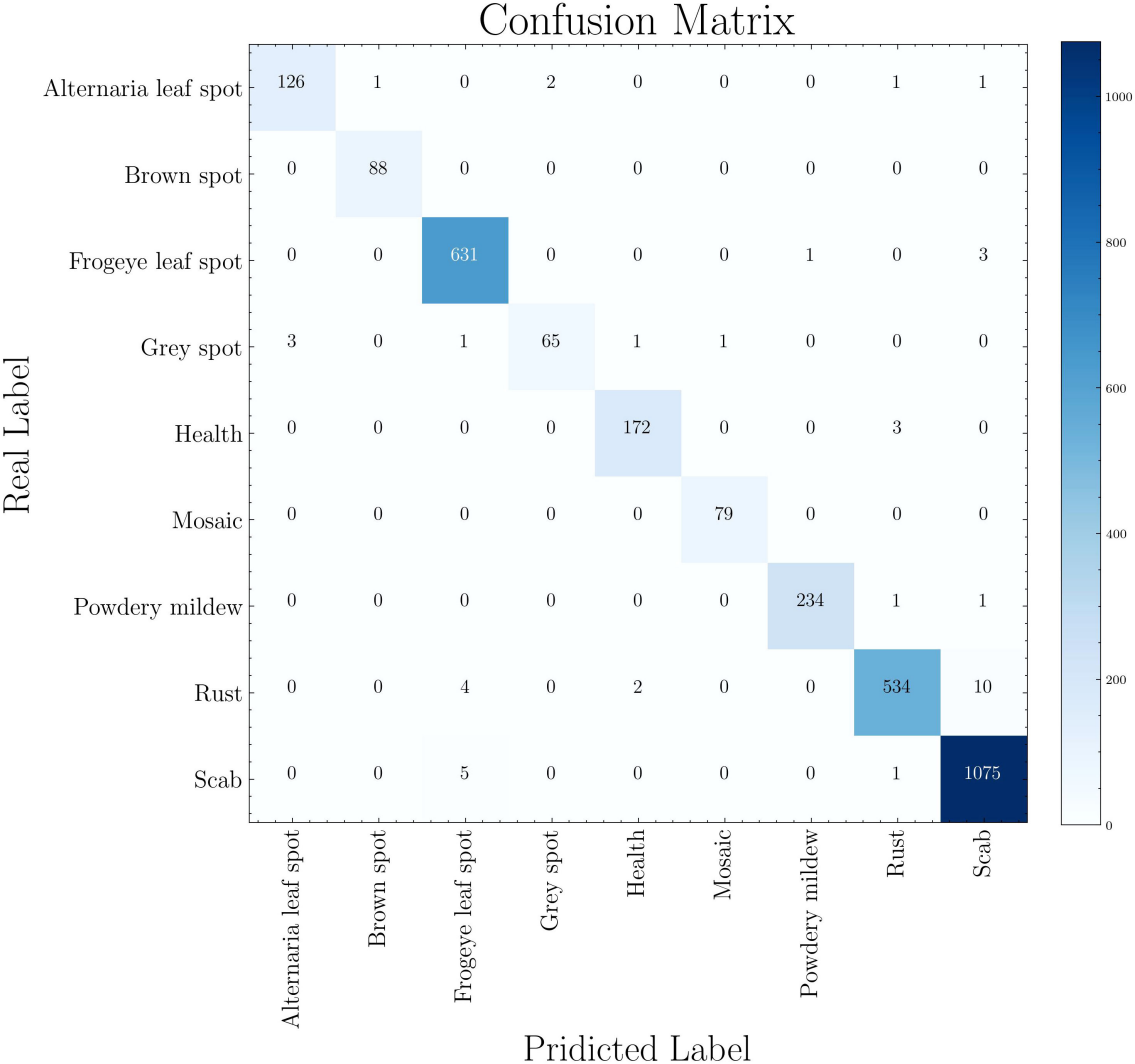
Confusion matrix of disease classification.

The confusion matrix of the experiment is shown in **Figure 6**, where the horizontal and vertical coordinates represent the disease predicted by the model and the real disease respectively. Therefore, when the prediction is consistent with the actual situation, the axis data of the matrix will be added by one. When the predicted disease is inconsistent with the actual disease, the increased value of the matrix appears in the nondiagonal region. Take ‘Rust’ as an example, 534 cases of Rust were accurately identified, but 4 cases were misdiagnosed as Frogeye, 2 cases were misdiagnosed as health, and 10 cases were misdiagnosed as Scab. The 10 misdiagnosed cases were also the most common in the model, due to the similarity in size and color between rust and scab. Next, we want to further modify the model to better distinguish between the two diseases.

### 4.3 ROC

**Figure 7:**
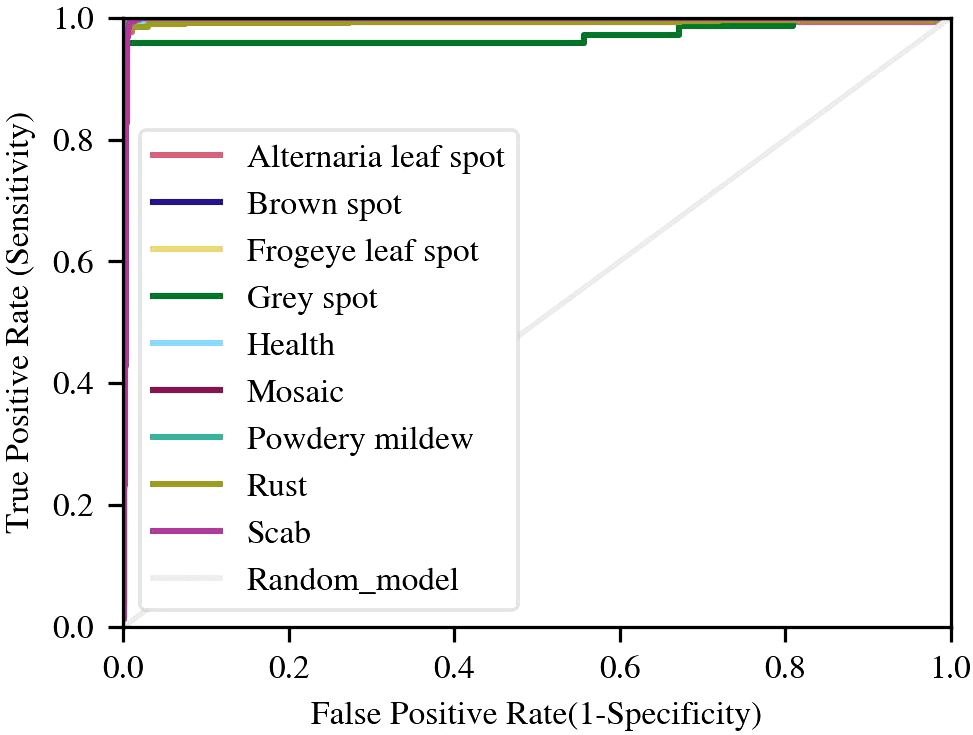
ROC curves of disease samples.

We have depicted the Receiver Operating Characteristic (ROC) curve of each disease, as shown in **Figure 7**. It should be noted that the true positive rate of various diseases is high, resulting in a very steep ROC curve. The curve of Gery spot is different from several other diseases, as it initially reaches around 0.95. When the false positive rate reaches over 0.6, the true positive rate further increases to over 0.98. The steep ROC curve shows that the model can distinguish various diseases very well. In contrast, the ROC of general models is only diagonal.

### 4.4 Comparison with other attention mechanisms

**Figure 8:**
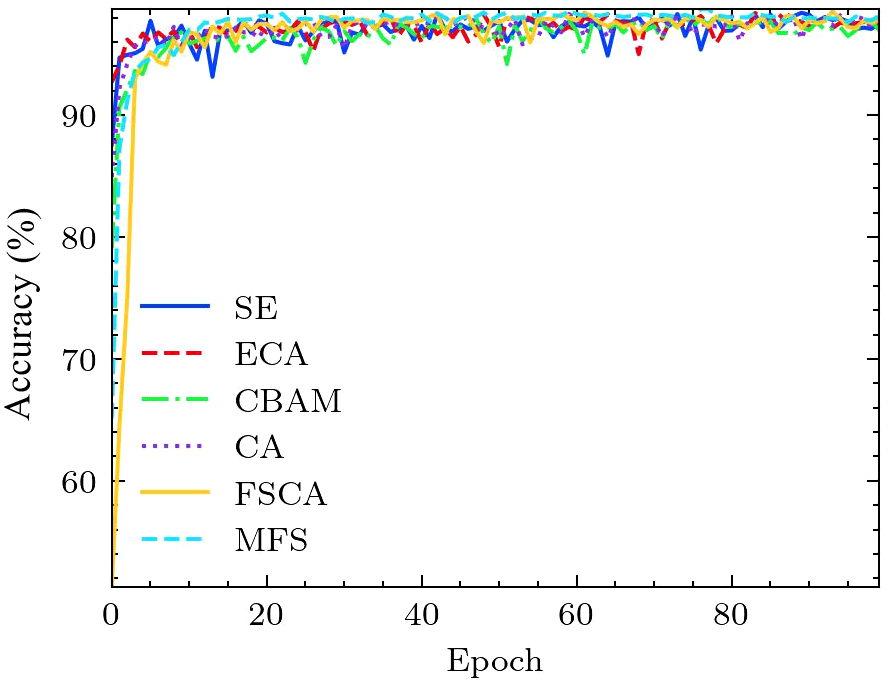
Comparison of accuracy curves for different attention mechanisms.

In order to visually display the impact of different attention mechanisms, we calculated and compared the accuracy of different attention mechanisms (SE, ECA, CBAM, CA, FSCA, MFS) within the MobileNet v3 framework. As shown in **Figure 8**, our proposed FSCA attention mechanism and combined multi-scale MFS attention mechanism grow rapidly with epochs but are slightly slower compared to other types. But when the epoch increases to 20, their stability and maximum value are the best. In contrast, the fluctuation amplitude of other attention mechanisms is relatively large, while the accuracy of the MFS and the FSCA mechanism fluctuates at the highest point, demonstrating special stability.

### 4.5 Comparison with other CNNs

**Figure 9:**
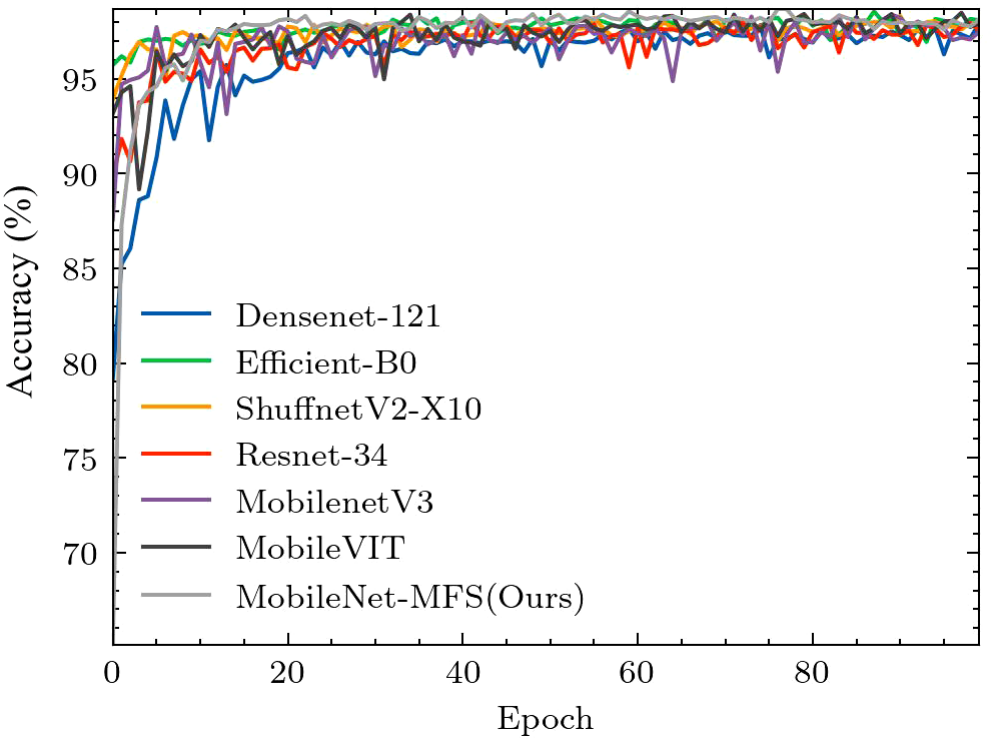
Comparison of accuracy curves for different models.

The accuracy of different CNNs and MobileNet-MFS are also compared. As shown in **Figure 9**, the light gray curve represents the accuracy curve of MobileNet-MFS. Compared with other models, it also rises very quickly and gradually reaches its high-level platform after 20 epochs. At the 28th epoch, MobileNet-MFS has an accuracy of around 98%, which is better than other models at the same epoch. Finally, when the epoch reaches 75, the MobileNet-MFS reaches its maximum accuracy of 98.7%, surpassing all other models.

In order to comprehensively compare our model with other classic models, we calculated several indicators such as precision, recall, f1 score, and AUC. These indicators can measure the model’s capabilities from different aspects. From **Table 2**, we can note that the MobileNet-MFS has the highest metrics in precision, recall and f1-score. However, in terms of AUC, it is not as good as a group of models such as EfficientNet-B0 and MobileNet-VIT.

**Table 2:** Precision, Recall, F1-Score and AUC for different models.

Model	Precision	Recall	F1-Score	AUC
MobileNetV3	0.982257	0.982272	0.982245	0.996483
Densenet121	0.978340	0.978332	0.978258	0.998184
EfficientB0	0.985624	0.985555	0.985524	**0.998827**
ShuffnetV2 X10	0.981947	0.981944	0.981842	0.998230
Resnet34	0.979438	0.979317	0.979282	0.997757
MobileVIT	0.984214	0.984242	0.984185	0.998727
MobileNet-MFS	**0.986198**	**0.986211**	**0.986156**	0.996105

Regarding the comparison of model performance, in addition to the above indicators, it is also necessary to consider the computational resources used by the models. MobileNet-MFS is based on MobileNet v3 and belongs to a lightweight CNN. The lightweight of the model will help it be applied to a wider range of scenarios. In addition, the computational complexity of the model is also a very important indicator, and the FLOPs provide an effective method to measure the computational complexity of the model. The indicators provided in **Table 3** help us measure various aspects of the model more comprehensively. Taking into account parameter counts, memory size, and FLOPs counts, The MobileNet-MFS has more advantages over EfficientNet-B0, ResNet-34, and DenseNet-121, consuming slightly more computing resources than MobileNet v3, but not as streamlined as ShuffleNet v2.

**Table 3:** Comparison of operational and parameter performance among different models.

Model	TOP-1 Accuracy (%)	Parameters Count (Millions)	Memory Size (MB)	FLOPs Count (MFLOPs)
MobileNet-MFS	98.69	4.96	51.30	251.94
MobileNetV3	98.39	4.21	50.39	226.44
Densenet121	97.90	6.96	147.10	2881.60
EfficientB0	98.56	4.02	79.40	398.03
ShuffnetV2 X10	98.33	1.26	20.84	149.58
Resnet34	98.09	21.29	37.61	3673.72
MobileVIT	98.49	1.94	–	743.48

In summary, through the comparison of various indicators, parameter quantities, and computational complexity, we can conclude that although many excellent models have emerged for image classification, MobileNet-MFS is still a state-of-the-art model.

## 5 Discussions

**Figure 10:**
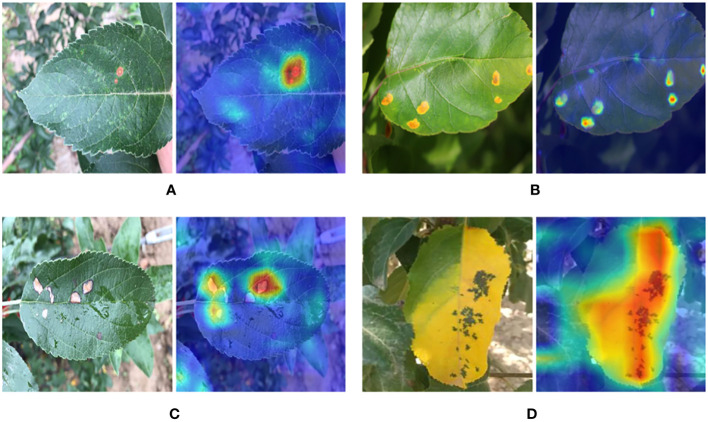
Heat map display of feature extraction of leaf disease sites: **(A)** Alternaria leaf spot **(B)** Rust **(C)** Grey spot **(D)** Brown spot.

Finally, we utilized Gradient-weighted Class Activation Mapping (GRAD-CAM) to extract network recognition feature maps of images. Through these feature maps, we can more intuitively see the model’s recognition of image features. As shown in **Figure 10A**, the Alternaria leaf spot on the leaf is very well and directly identified. From **Figures 10B, C**, it should be noted that the lesion areas on the Rust and Gray spot leaves with multiple spots have also been simultaneously observed, without any omissions or misjudgments. As shown in **Figure 10D**, the large area of yellow on the brown spot was well captured by our model, and the spots on the brown spot were also given special attention. These figures demonstrate the model’s excellent feature capture ability.

**Figure 11:**
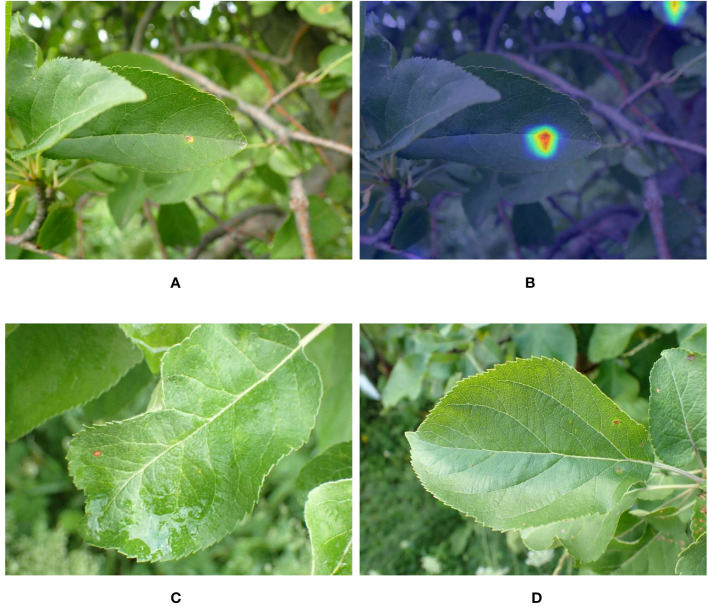
**(A)** Leaves with Rust disease. **(B)** Heat map of feature extraction of the Rust lesion site. **(C, D)** Mistakenly identified leaves with Frogeye disease.

The error case of MobileNet-MFS is also checked, and these images are selected from the library. As shown in **Figures 11A, B**, the Rust lesion can be accurately captured by our model. However, the leaves in **Figures 11C, D** with Frogeyes disease were mistakenly identified by the model as Rust-infected leaves. It can be deduced that these erroneous cases are due to the many similarities in the characteristics of these two diseases, and this discrimination error should be very difficult for CNNs.

From the perspective of incorrect images, it is actually difficult for the human eye to distinguish between the two situations. We cannot rule out that the database itself may still have misclassification in some cases. Without proper management, the error rate of the human eye itself is within the range of 5% -10%. If artificial intelligence is well-trained, it can surpass human recognition ability. Therefore, considering randomness, we believe that certain errors are inevitable.

Simply comparing accuracy, our work is inferior to some recent work. However, on the one hand, our dataset differs from theirs, as a large proportion of the images in our dataset are collected from the natural environment. On the other hand, the parameters and operation time of our model are also different. Although 98.7% is a high-level score for the classification of leaf diseases, the images in our dataset have been well processed, so they cannot fully restore the real usage scenarios. We have not yet processed images taken in orchard environment, therefore it is the weakness of our work. Our next step is to develop a network that can process drone and robot camera images, remove unclear and messy backgrounds, and make accurate classifications on mobile devices.

## 6 Conclusions

The identification of apple leaf diseases is very difficult, thanks to the development of deep learning, a series of models have shown great achievement in identifying leaf diseases. On the basis of these works, we have improved MobileNet v3 by modifying its attention mechanism, taking into account the influence of dimension and space. At the same time, we have added a multi-scale feature extraction module to further improve the performance of the network. By comparing with similar models, we found that our proposed MobileNet-MFS showed the best performance in terms of accuracy and stability. This also indicates that our proposed attention mechanism and multi-scale module have effectively improved the feature capture ability of the model for leaf diseases, and there is also hope for their application in other aspects. We also calculated the ROC and confusion matrix of the model, which shows that the model is very good at resolving various diseases. Finally, we reviewed the feature extraction graph of the model through GRAD-CAM and analyzed the error cases. Compared to previous models, the model is more efficient mainly due to the mutual cooperation of two aspects. FSCA and multi-scale respectively increase the model’s feature discovery ability and the implementation of more scale features, both of which are crucial for getting more accurate classifications. This work indicates that the MobileNet-MFS is a very effective model for distinguishing apple leaf diseases, and the FSCA attention mechanism used in this model is also worthy of further application in other scenarios.

## Data availability statement

The original contributions presented in the study are included in the article/supplementary material. Further inquiries can be directed to the corresponding author.

## Author contributions

HC: Methodology, Software, Visualization, Writing – review & editing. HL: Investigation, Visualization, Writing – original draft.
